# Gait Analysis as a Measure of Physical Performance in Older Adults with Bilateral Knee Osteoarthritis

**DOI:** 10.3390/medicina61122118

**Published:** 2025-11-28

**Authors:** Kamal Constantin Kamal, Adina Maria Kamal, Diana Kamal, Ovidiu Fugaru, Daniela Matei, Magdalena Rodica Trăistaru

**Affiliations:** 1 Department of Family Medicine, University of Medicine and Pharmacy Craiova, 200349 Craiova, Romania; 2 Department of Internal Medicine, University of Medicine and Pharmacy Craiova, 200349 Craiova, Romania; 3 Department of Physical Rehabilitation, Filantropia Hospital Craiova, 200516 Craiova, Romaniaovidiufugaru4@gmail.com (O.F.); 4 Faculty of Nursing, University of Medicine and Pharmacy Craiova, 200349 Craiova, Romania

**Keywords:** older adults, gait analysis, quadriceps ultrasound, knee osteoarthritis, physical performance

## Abstract

*Background and Objectives.* Bilateral knee osteoarthritis (KOA) in older patients compromises mobility and quality of life. Gait analysis provides objective, reproducible measures of physical performance. This study evaluated the integration of gait analysis for longitudinal monitoring and personalized rehabilitation, while ultrasound was performed only at baseline for characterization and did not inform adjustments to the interventions. *Materials and Methods.* We conducted a 6-week, randomized, parallel-group clinical trial including 130 participants aged ≥65 years. Patients were randomized to a Study Group (SG, *n* = 64) receiving 10 sessions of a comprehensive rehabilitation program (conventional measures plus gait training) or a Control Group (CG, *n* = 66) receiving 10 sessions of conventional rehabilitation (electrotherapy with magnetodiaflux, TENS, therapeutic ultrasound, low-intensity laser, plus standard kinesitherapy). Interventions were delivered daily, 5 days/week, over 2 consecutive weeks. Gait outcomes (BTS G-WALK/G-SENSOR 2) included TUG, Symmetry Index, 6MWD, and cadence; functional outcomes included VAS pain, WOMAC, and Lequesne Index. Quadriceps morphology was assessed sonographically, and a height-normalized quadriceps thickness index (QHNI) was calculated. *Results.* Of 130 randomized, 112 completed (93.3%). Compared with control, the intervention produced significant, clinically meaningful improvements: 6MWD increased by approximately 59 m, cadence by ~9 steps/min; TUG improved by ~2.6 s; gait symmetry by ~4–5 points; VAS pain decreased by ~1.7 points; WOMAC total by ~8.5 points; and Lequesne Index by ~2 points (all *p* < 0.001). QHNI showed no significant association with anthropometrics and performance measures, indicating limited value as a linear predictor at baseline; nonlinear models or subgroup analyses may be warranted. *Conclusions.* Both neuromuscular-focused rehabilitation and the conventional program improved gait and clinical outcomes. Integrating gait analysis with ultrasound evaluation enables comprehensive monitoring and supports personalized interventions to reduce joint loading and optimize gait mechanics in older patients with bilateral KOA.

## 1. Introduction

Knee osteoarthritis (KOA), the most common site of osteoarthritis, is a chronic, disabling, and multifactorial condition that affects the entire knee joint [1]. Its prevalence has increased substantially over the last decade, and KOA ranks among the leading contributors to years lived with disability worldwide [2,3]. It is highly prevalent in older adults [4] and is a major cause of pain, impaired mobility, and loss of independence, with a higher burden observed in women after the age of 60 [1]. Beyond personal suffering, KOA imposes considerable economic costs due to medication use, hospitalizations, and joint replacement surgeries [5,6].

The quadriceps is central to knee stability, shock absorption, and gait propulsion; stronger muscles better support alignment during weight-bearing and enhance postural stability [7,8,9,10], while reduced thickness—particularly in the rectus femoris and vastus medialis, measurable noninvasively via ultrasound or MRI—correlates with pain, radiographic severity, and functional limitation, and contributes to anterolateral instability and dynamic malalignment [11,12]. Accordingly, quadriceps muscle thickness serves as a practical biomarker for risk stratification and monitoring the response to conservative care, and quadriceps function (strength and neuromuscular control) remains a key determinant of physical performance in older adults with knee osteoarthritis [13,14].

KOA can be considered a mechanically driven disease with a significant functional impact on the gait pattern, one of the important functional limitations in this lower-limb disorder [15,16,17,18]. Gait dysfunction in KOA is an important subject for the physical medicine and rehabilitation team [19,20]. Before gait retraining, it is essential to perform a gait analysis of the patient and to evaluate their physical performance.

Gait analysis, therefore, is emerging as a key tool to quantify physical performance and guide management in bilateral KOA among older adults [21]. Spatiotemporal parameters (gait speed, cadence, step length), kinematic patterns (sagittal knee excursion, frontal plane alignment), and kinetic variables (knee extensor moments) can reveal the functional consequences of quadriceps atrophy and weakness, including reduced push-off, cautious gait strategies, asymmetries, and compensations at the hip and ankle [22]. In the context of bilateral KOA—where symmetrical involvement can mask side-to-side comparisons and amplify global mobility constraints—gait analysis offers an especially valuable lens for evaluating disease impact and tailoring therapy [23,24]. Over the last decade, there has been growing interest in integrating assessments of quadriceps thickness and function with gait-derived performance metrics. By doing so, clinicians and researchers can better elucidate the mechanisms linking muscle deficits to mobility limitations, optimize nonoperative care, and refine criteria for surgical referral [25].

In this article, we consider gait analysis as a measure of physical performance in the management of bilateral knee osteoarthritis in older adults, emphasizing its implications for prognosis, personalized rehabilitation, and outcome monitoring.

First, we established a height-normalized quadriceps thickness index (QHNI) to capture the muscle component relevant to lower-limb function, independent of global anthropometric variation. QHNI reflects knee extensor potential—a major determinant of stability and gait spatiotemporal parameters—and enables accurate inter-individual comparisons by controlling for height. By normalizing, the index reduces confounding due to body habitus and improves sensitivity to clinically meaningful changes compared with raw thickness measures.

Second, we assessed gait parameters in bilateral KOA older adults and correlations between gait parameters (gait function tests or performance-based measures) and pain and functional status, pre- and post-rehabilitation program, using the G-walk sensor produced by the BTS company [26]. Performance-based measures are defined as assessed or observed measures of tasks and are usually assessed by timing, counting, or distance methods. These parameters evaluate what an individual can do rather than what the individual perceives they can do [27], for example, walking, standing, and climbing. In patients with KOA, these tests have been widely used as objective assessments and are recommended by the Osteoarthritis Research Society International (OARSI) for clinical application [28]. We hypothesized that physical performance would have a complete correlation with pain, QHNI, and functional status, and post-rehabilitation physical performance-based measures would be better than prehabilitation.

## 2. Materials and Methods

### 2.1. Ethical Approval

Before being included in the study, the details of the present research were explained to the KOA patients. Written informed consent was obtained from each patient. The protocol was conducted in accordance with the Declaration of Helsinki and Good Clinical Practices and was approved by the local independent Ethics Committee of Filantropia Hospital Craiova (approval no. 19689/14 August 2025).

### 2.2. Study Design

Our prospective randomized study was conducted in the Department of Physical Medicine and Rehabilitation, Filantropia Hospital, Craiova, over a period of two months, from 15 August 2025 to 14 October 2025.

Based on an initial screening of 160 consecutive patients presenting with bilateral KOA, 130 individuals met the preliminary inclusion criteria (ACR—American College of Rheumatology criteria for KOA) [29]. Randomization was made by an independent physician, taking into consideration the patient’s consent for gait training. After initial assessment, a total of 130 patients were randomly divided into two groups: Study Group (SG = 64 patients) received 10 sessions of the complete rehabilitation program, and Control Group (CG = 66 patients) received 10 sessions of the conventional rehabilitation program.

Both of these rehabilitation programs were performed daily, 5 days/week, for 2 weeks. The conventional rehabilitation program included electrotherapy measures (magnetodiaflux, transcutaneous nerve stimulation, ultrasound, and low-intensity laser treatment) and standard kinetic measures. The complete rehabilitation program included conventional measures and gait training.

**Figure 1:**
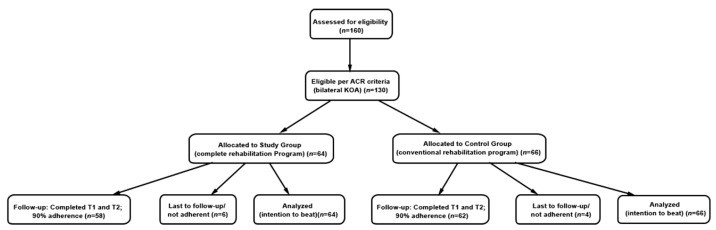
Diagram of our study.

Only patients who completed both T1 (initial) and T2 (after 6 weeks) evaluations, attended at least 80% of rehabilitation sessions, and had valid datasets were included in the final analysis. We scheduled T2 at 4 weeks post-intervention to assess the durability of effects and for logistical reasons. This included 58 participants in the SG (90%) and 62 in the CG (93%). Figure 1 presents the study flow diagram. 

The inclusion criteria taken into account when designing the groups were as follows:

- Patients older than 65 years, age ranging between 65 and 80 years, diagnosed with KOA according to ACR criteria, and are also accepted in our country;
- Absence of knee injuries for at least 6 months before;
- At least 3 years of disease progression;
- Absence of major disturbances in the frontal plane alignment of the knee;
- Painful knee for a period of 48 h after physical activity;
- Compliance with physical exercise during the healthcare program;
- Patients with other co-morbidities, but well controlled, like dyslipidemia, arterial hypertension, and type II diabetes mellitus.

Patients will be excluded if they have a history of knee replacement, unstable medical conditions preventing them from participating in the rehabilitation programs, and/or neurological or any other conditions affecting the strength or function of their lower limbs.

Evaluation of patients was carried out in two stages—initial (T1) and 6 weeks after the initial assessment (T2), more precisely, 4 weeks after the completion of the rehabilitation program.

### 2.3. Patients’ Assessment

Each study visit (T1 and T2) consisted of a clinical evaluation, measurement of vital signs, physical performance-based measures, and a functional assessment.

Laboratory testing and imaging evaluation were performed only at the initial assessment (T1) for all patients.

After careful anamnesis (age, comorbid diseases), the ***clinical assessment*** included the following:

- General physical examination (body mass index—BMI);
- Musculoskeletal and neurological examination—somatoscopic exam, assessment of the range of motion, and manual muscle testing of the lower-limb muscles;
- Balance and gait examination.

First, we conducted ***standard laboratory tests*** (screening, inflammation tests—fibrinogen and C-reactive protein). Blood samples for laboratory analyses were carefully obtained from the antecubital veins of all patients after an overnight fast, under aseptic conditions. Tests were performed using three automated blood analyzers (MINDRAY BC-6800 (Mindray, Shenzhen, China) for the complete blood count, ARCHITECT C4000 (Abbot Laboratories, North Chicago, IL, USA) for biochemical analysis, and SYSMEX CS-2500 (Sysmex, Kobe, Japan) for fibrinogen).

***Imaging evaluation*** of the patients included ***radiographic examination*** of the knees and ultrasound assessment of the quadriceps muscle.

The Kellgren and Lawrence Radiographic KOA (KL) scoring was interpreted by a physician based on recent weight-bearing, anterior–posterior X-rays of the tibiofemoral joint for both knees without knowledge of the clinical conditions.

**Figure 2:**
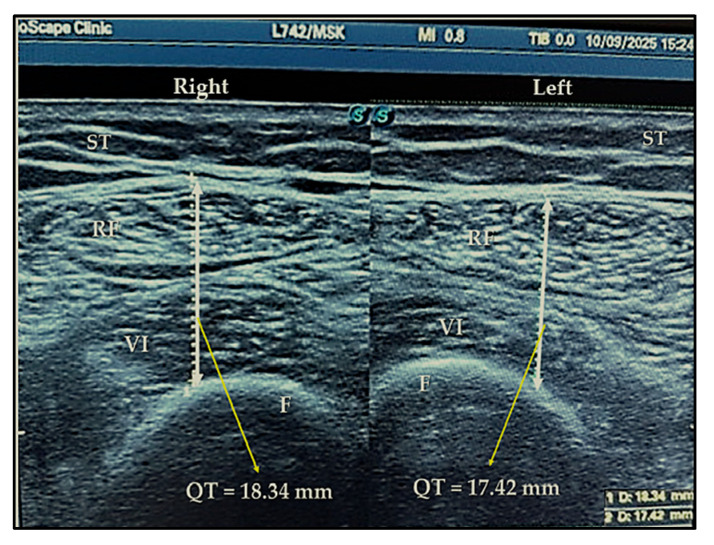
Thigh muscle thickness measurement. QT = quadriceps thickness; RF = rectus femoris; VI = vastus intermedius; F = femur; ST = subcutaneous tissue.

***An ultrasound examination*** was made with the patient in a supine position, with flexed, relaxed lower limbs and supinated upper limbs. We selected muscle thickness for feasibility and reproducibility in our setting. The exam is conducted by a qualified sonographer. The examination was carried out on a one-on-one basis in a private setting where only the patient and the sonographer were present. We respected the protocol described by Tillquist et al. using a 7.5 MHz linear transducer connected to an ultrasound device (SonoScape S9Pro, SonoScape Medical Corp., Shenzhen, China) [30]. Transverse ultrasound section made at the two-thirds (“two-thirds” site) and the midpoint (“midpoint” site) of the length between the anterior superior iliac spine (ASIS) and the upper border of the patella. Both measurement sites were determined using a measuring tape and analyzed in order to find the most reliable point. After identifying the muscle tissue, the thickness of the quadriceps muscle was obtained by measuring the distance between the cortex of the femur and the most superficial muscular fascia (Figure 2). Measurements were performed by applying maximal compression on the ultrasound probe without inflicting pain, in order to prevent the underestimation of muscle wasting linked to subcutaneous edema. We calculated a height-normalized quadriceps thickness index (QHNI) as the sum of both the muscle’s right thickness (RT) and left thickness (LT) in cm/height^2^ (m^2^). This index is a reasonable, practical biomarker for monitoring quadriceps morphology over time and can support risk stratification in elderly patients with bilateral KOA [31].

***Functional evaluation*** included the following:

- The VAS—Visual Analog Scale (from 0 to 10, 0 = absence of pain and 10 = maximum pain score; other values between 0 and 10 are directly proportional to the individual pain threshold) [32];
- The Western Ontario and McMaster Universities Osteoarthritis Index (WOMAC) contains 24 specific questions divided into three domains: pain (P-WOMAC; 5 items), stiffness (S-WOMAC; 2 items), and physical function (PF-WOMAC; 17 items). The score of each question ranges from 0 to 4. The 0 score is equivalent to maximal functional status, and a high score of 96 indicates a minimum status, with high disruption in day-to-day tasks [33];
- The Lequesne Functional Index (a 10-item questionnaire) 24 is the minimum, the worst outcomes, and 0 is indicative of less functional impairment or maximum functional status. Lower-limb dysfunction is grouped in 0 (none), 4 (mild), 5–7 (moderate), 8–10 (severe), 11–13 (very severe), and more than 14 (extremely severe, limiting and dysfunctional) [34].

These two scales were used for determining the effect the disease has on performing daily living activities.

***Physical performance-based measures*** were assessed using the gait analysis. We used a wireless system—BTS G-WALK (BTS Bioengineering Corp., Garbagnate Milanese, Italy)/BTS G-SENSOR 2—consisting of an inertial sensor, composed of a tri-axial accelerometer, a magnetic sensor, and a tri-axial gyroscope, which, when worn by the patient, allows for a functional gait analysis [35].

Gait analysis with G-WALK was conducted under standardized conditions: participants walked barefoot wearing only socks, without assistive devices, on a straight, smooth indoor walkway with controlled lighting and temperature. The protocol included two familiarization trials followed by the test trial; the sensor was mounted at the L5 level according to the manufacturer’s instructions and secured with an elastic belt.

This system can be connected with a force platform (P-WALK), where the gait information is measured through pressure sensors and ground reaction force sensors (GRF), which measure the force exerted by the patient’s feet on the floor when they walk. These parameters were as follows:

- Timed Up-and-Go (TUG) test—patients stood up from an armchair, walked at a safe and comfortable pace to a line 3 m away, crossed the line, turned, and returned to a sitting position in the chair; none of the patients used a walking aid, and the time to complete the task was recorded.
- Symmetry index (SI)—for the patient’s ability to have an identical model of acceleration and deceleration of their center of mass regardless of the side of the gait cycle.
- Six Minutes Walking Test (6 MWT)—“walking distance” (6 MWD in meters) and “average cadence” (steps/min).

### 2.4. Rehabilitation Program

The healthcare objectives were as follows:

- Painful status control;
- Regaining stability and mobility of the knee and restoring balance to the muscle groups serving the entire “knee” complex;
- Correcting the abnormal walking scheme;
- Regaining motor control and optimal knee function.

The performed measures in both groups of patients were the following:

- Non-pharmacological measures—educational, dietary, and hygienic;
- Pharmacological measures—analgesics and chondroprotective drugs;
- Physical therapy—magnetic therapy, transcutaneous nerve stimulation (TENS), ultrasound, and low-intensity laser treatment (Table 1);
- Kinetic training—all patients received conventional kinetic therapy. For the SG patients, the kinetic program included gait training measures (Table 2). At discharge, all patients were advised to continue the learned kinetic exercises at home.

### 2.5. Statistical Analysis

Observational studies are often limited by imbalances in known and unknown confounders. To minimize potential confounding effects when evaluating variables before and after treatment, we performed statistical analyses using SPSS, version 26.0 (IBM Corp., Armonk, NY, USA). In addition, we used Python (GenAI), version 3.10, for data preprocessing and supplementary visualizations.

The tabulated data are coherent (numeric), so parametric tests were used where their assumptions were met. Quantitative variables are expressed as means ± standard deviations. We used Levene’s test to assess the homogeneity of variances, whose results are less affected by unequal group sizes. After applying the Shapiro–Wilk normality test, most variables were found to be non-normally distributed. Therefore, for within-group comparisons (baseline vs. post), the Wilcoxon signed-rank test was applied. For between-group comparisons at the same time point (baseline 1 or post 2), the Mann–Whitney U test was used. For transparency, we reported effect sizes appropriate to each test: r = Z/√*n* for the Wilcoxon test; Cohen’s d for parametric independent analyses; and r = Z/√*n* for the Mann–Whitney U test.

Chi-square tests of independence were applied to compare SG vs. CG on categorical variables: sex distribution and residence (urban vs. rural).

To evaluate relationships among continuous variables, we computed Pearson correlation coefficients and presented the results in correlation matrices. Statistical significance was set at *p* < 0.05.

The sample sizes used in the analysis (approximately 58–62 per group; similar paired *n* for within-group comparisons) ensure valid and interpretable results, with adequate power to detect small-to-moderate effects and high power for moderate-to-large effects.

We used AI- Python Programming, version 3.10, for data preprocessing and supplementary visualizations.

## 3. Results

### 3.1. Anthropometric Data

In SG (*n* = 58), women significantly predominate, comprising over 50%. The urban vs. rural distribution does not deviate significantly from 50:50 (Table 3).

In CG (*n* = 62), women are more numerous, but not significantly at the 5% level. The urban vs. rural distribution is approximately 50:50 (Table 4).

The distribution by gender and residence (urban vs. rural) was also similar between groups (chi-square tests, *p* = 0.625 for sex and *p* = 0.347 for residence).

**Figure 3:**
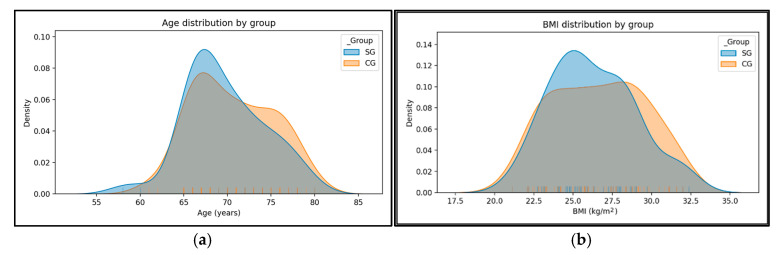
Age distribution by group (**a**). BMI distribution by group (**b**).

In total, the SG and CG were comparable in age and body mass index (BMI). Independent-samples *t*-tests indicated no significant differences between groups for age (*p* = 0.288) or BMI (*p* = 0.481). Both SG and CG show bell-shaped distributions centered in the late 1960s to early 1970s, with substantial overlap. CG has a slightly right-shifted peak and a marginally heavier right tail (more participants in the older range), but the difference is small (Figure 3a). The BMI curves for SG and CG overlap broadly around the mid-20s. CG shows a subtle rightward shift and a somewhat heavier right tail (higher-BMI values), while SG’s density peaks slightly lower. Differences are modest; both groups cluster in the mid- to upper-20s range without a pronounced gap (Figure 3b).

Within-group associations showed no significant correlation between age and BMI in either SG or CG. Gender was strongly associated with BMI in both groups (point-biserial correlation, *p* < 0.001), with BMI differing between men and women, while gender did not relate to age. Residence (urban vs. rural) was not associated with age in either group; a weak association with BMI was observed only in CG.

**Figure 4:**
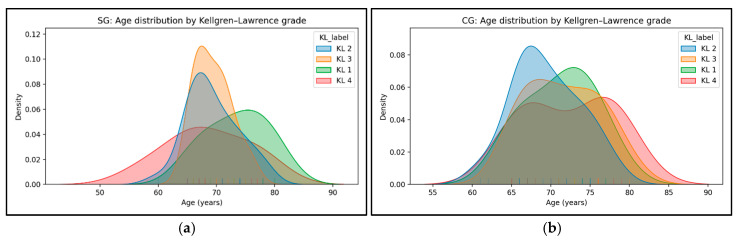
Age distribution by Kellgren–Lawrence grade for study group (**a**) and for control group (**b**).

In both groups, patients with mild and moderate forms of knee osteoarthritis predominated. In the SG, there were six cases with KL grade 1, twenty-six with grade 2, twenty with grade 3, and six with grade 4. The age densities for KL 1–4 largely overlap and center in the late 1960s to early 1970s. Higher KL grades show a slight rightward shift and broader spread, suggesting somewhat older ages at higher severity, but separation remains modest. Overall, increases in KL appear associated with small, incremental age shifts rather than distinct strata (Figure 4a). In the CG, there were six cases with KL grade 1, thirty with grade 2, eighteen with grade 3, and eight with grade 4. Similarly to SG, KL curves overlap substantially with peaks in the late 60s to early 70s. Higher KL grades lean toward older ages with mild widening of the tails. Figure 4b shows incremental rather than stepwise age differences across KL grades, for CG.

The mean values for all biochemical and inflammatory tests were included in the normal interval. These aspects permitted the application of the rehabilitation program in safe conditions.

We prescribed a rehabilitation program adapted to the individual’s clinical and functional status.

In the SG, we achieved better gait control through gait-specific training, whereas physiotherapy and a conventional kinesitherapy program were less effective for controlling the walking pattern.

### 3.2. Evolution of Parameters in the SG (Baseline to Post-Intervention)

Overall, the SG demonstrated robust and clinically meaningful improvements in walking capacity, mobility, gait parameters, pain, and disability from baseline to follow-up.

**Figure 5:**
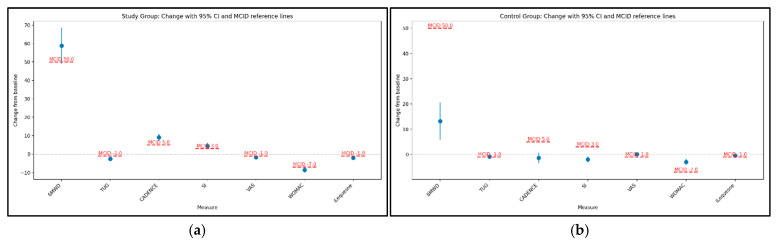
Change in parameters with 95% confidence intervals and MCID reference lines for study group (**a**) and for control group (**b**).

We compared the observed mean changes and their 95% CIs to commonly cited MCID thresholds for each parameter. When the entire CI exceeds the MCID, the improvement is robustly clinically meaningful. The study group achieved clinically meaningful improvements across outcomes relative to established MCID thresholds. Mean changes in 6MWD (+58.7 m, 95% CI 49.3 to 68.2) and TUG (−2.61 s, 95% CI −3.11 to −2.11) exceeded typical MCIDs (≈30–50 m and ≈0.8–1.4 s, respectively). Pain decreased beyond MCID on VAS (−1.74 points, 95% CI −1.94 to −1.55), and disability improved to a clinically important extent on WOMAC (−8.52 points, 95% CI −9.74 to −7.30) and Lequesne (−1.98 points, 95% CI −2.24 to −1.73). Cadence (+9.12 steps/min, 95% CI 7.59 to 10.65) and gait symmetry (+4.42 points, 95% CI 3.12 to 5.72) also demonstrated improvements exceeding pragmatic thresholds, indicating meaningful functional gains (Figure 5a).

The subgroup comparisons showed no statistically significant differences between women and men across physical performance (*p* > 0.05, for all parameters) and functional outcomes at baseline (*p* = 0.108, for VAS, *p* = 0.176 for WOMAC, *p* = 0.285 for Lequesne Index, between gender); urban vs. rural showed similarly small differences (e.g., 6MWD baseline 335.15 ± 63.75 vs. 334.50 ± 53.41 m; VAS baseline 6.65 ± 1.13 vs. 7.08 ± 1.02; both *p* > 0.05). At the final assessment, there were no statistically significant differences between women and men, nor between urban and rural subgroups, across all parameters.

### 3.3. Evolution of Parameters in the CG (Baseline to Post-Intervention)

In the CG, within-group changes from baseline were modest and predominantly below clinically meaningful thresholds.

In the control group, changes generally fell below MCID thresholds: the increase in 6MWD (+13 m; 95% CI 6.03 to 20.33; Wilcoxon *p* < 0.001) and the decrease in TUG (−0.84 s; 95% CI −1.26 to −0.42; Wilcoxon *p* < 0.001) do not typically reach MCID (≈30–50 m and ≈0.8–1.4 s, respectively, with TUG at the lower bound), and shifts in cadence, SI, VAS, WOMAC, and Lequesne were small, indicating modest clinical impact despite some statistical significance (Figure 5b).

CG shows a subgroup difference only at baseline for the Lequesne index (women > men, *p* ≈ 0.012). At the final assessment, there were no significant differences between women and men or between urban and rural participants for any evaluated parameter.

### 3.4. Between-Group Comparisons

At baseline, the SG showed a longer 6MWD (334.9 ± 59.2 m vs. 316.3 ± 43.9 m; mean difference 18.6 m, 95% CI −0.16 to 36.76) and a lower TUG (20.13 ± 4.91 s vs. 24.45 ± 4.56 s; mean difference −4.32 s, 95% CI −5.99 to −2.61; *p* < 0.001; r = 0.453), with small or non-significant differences for cadence and VAS. Conversely, SG had higher WOMAC at baseline (difference 6.97 points, 95% CI 4.44 to 9.39; *p* < 0.001) and lower SI (−3.77, 95% CI −6.40 to −1.21), indicating a heterogeneous starting profile across functional and symptom domains. These baseline imbalances were accounted for in post-treatment comparisons using a conceptual ANCOVA framework so that final contrasts reflect differences adjusted for baseline values.

**Figure 6:**
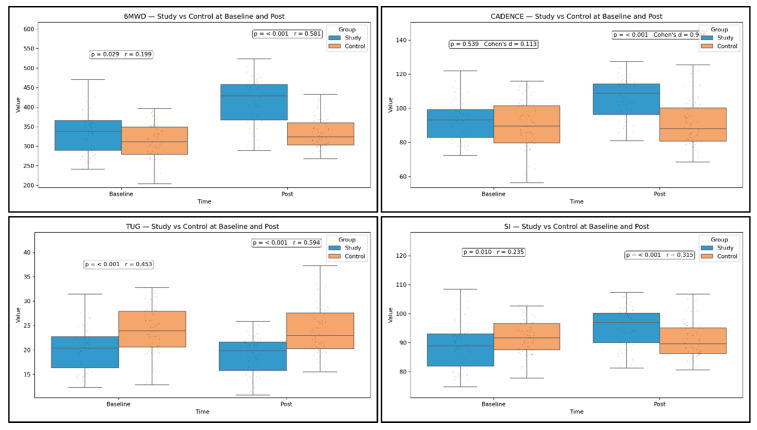
Between-group comparisons of physical performance parameters. Study vs. Control at Baseline and Post, with *p* and effect size.

Post-intervention, the SG outperformed the control group across functional and symptomatic outcomes with moderate-to-large effects. 6MWD was higher by 64.14 m (95% CI 45.62 to 82.08; *p* < 0.001; r = 0.581), and TUG was lower by 6.09 s (95% CI −7.67 to −4.47; *p* < 0.001; r = 0.594). Cadence increased more in the study group (a significant between-group difference), and SI improved in favor of the study group (Figure 6).

**Figure 7:**
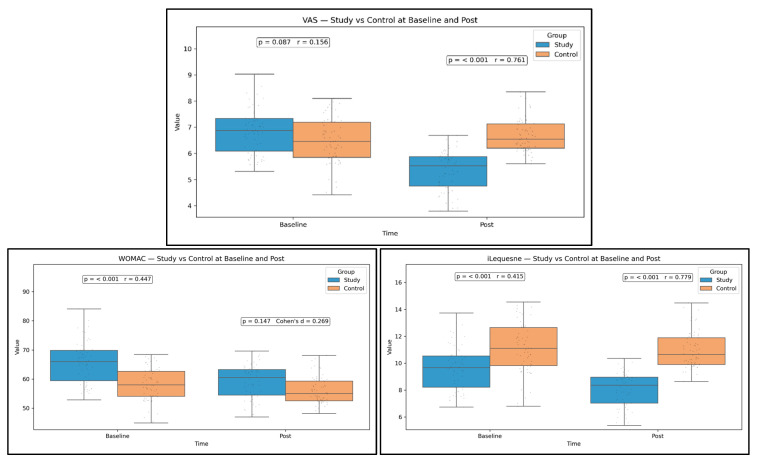
Functional parameters. Study vs. Control at Baseline and Post, with *p* and effect size.

The higher the walking cadence and the higher the symmetry index, the more optimal the gait pattern. The explanation is the gait-training program delivered to the SG. Pain (VAS) and disability indices (WOMAC, Lequesne Index) were significantly lower in the study group compared with controls at follow-up, with confidence intervals excluding zero and small-to-moderate effects depending on the measure (Figure 7). We did not include the WOMAC subscales, considering them non-essential clinical parameters for this study.

Overall (Table 5), the intervention delivered to the study group produced robust within-group improvements of large magnitude across walking capacity, mobility, spatiotemporal gait metrics, pain, and composite disability. Compared with usual care (control), the study group demonstrated superior post-intervention performance with clinically meaningful between-group differences, most notably for 6MWD and TUG.

The comprehensive rehabilitation program, including gait training, had a real impact on functional status.

### 3.5. Correlations Among the Studied Parameters at Baseline

We evaluated Pearson correlations for both groups, baseline, to explore linear relationships among functional, physical performance, and morphological (QMI) parameters, guiding covariate selection and clinical interpretation without implying causality.

**Figure 8:**
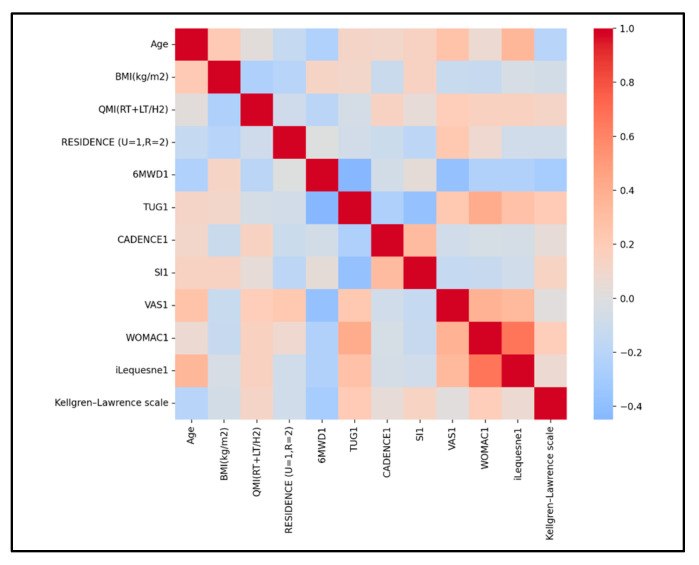
Selected parameters correlation heatmap for SG.

SG correlations (Figure 8). Significant correlations between functional and physical performance were as follows:

- 6MWD shows negative moderate associations with TUG VAS, and small with the Lequesne index, indicating that a greater walking capacity aligns with faster TUG, less pain, and slightly lower Lequesne disability.
- CADENCE is negatively associated with TUG (r = −0.335, small–moderate) and positively associated with 6MWD (r = 0.204, small), indicating that higher cadence aligns with faster TUG and slightly longer walking distance.
- TUG shows positive associations with both WOMAC (r = 0.410, moderate) and Lequesne index (r = 0.340, small–moderate), indicating that slower TUG aligns with greater disability on both scales.
- Lequesne Index shows positive associations with both VAS (r = 0.337, small–moderate) and WOMAC (r = 0.663, strong), indicating that higher pain and higher WOMAC scores align with greater Lequesne disability.

QMI shows only small associations across measures—slightly higher QMI among shorter participants (r ≈ −0.12) and weak links with 6MWD, TUG, CADENCE, and functional scores (VAS, WOMAC, Lequesne index; |r| < 0.2)—indicating that morphology captured by QMI relates weakly to pain and disability at Time 1. All these small QMI correlations with performance and symptoms suggest a limited role as a linear predictor at T1 or the need for nonlinear models/subgroup analyses. A lack of correlation between quadriceps thickness and functional/performance outcomes in KOA is plausible and commonly reported. Muscle function and performance depend on far more than size (thickness) alone.

Radiographic severity shows only small associations at baseline: modestly higher disability on Lequesne (r ≈ +0.20), essentially no link with WOMAC (r ≈ +0.04), a slight tendency toward shorter 6MWD1 (r ≈ −0.12), and weak relationships with pain (VAS1; |r| < 0.2).

**Figure 9:**
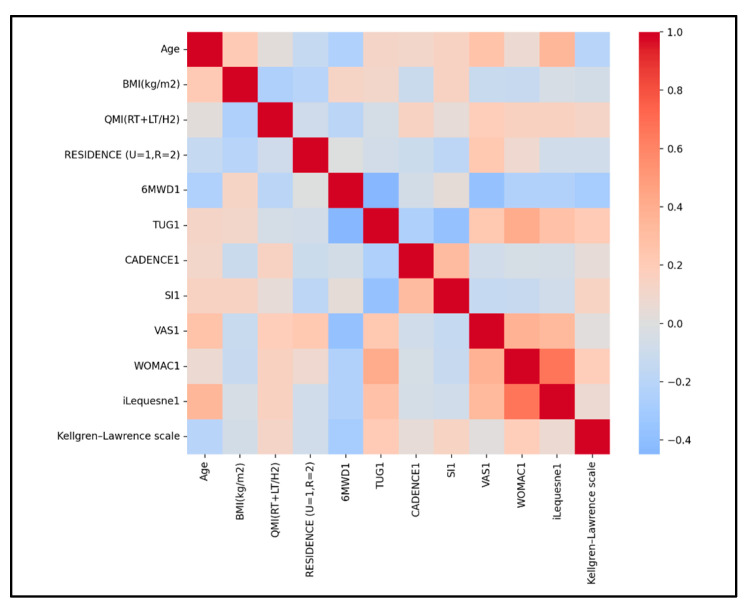
Correlation heatmap of selected parameters for CG.

For the CG at baseline, the key Pearson correlations (Figure 9) were as follows:

- 6MWD shows a small negative association with VAS (approx. r −0.13 to −0.26), is likely small and negative with WOMAC, and is small-to-moderately negative with Lequesne index, indicating that higher pain and disability relate to shorter walking distances.
- TUG shows a small positive association with VAS (r ≈ +0.13), and small-to-moderate positive associations with WOMAC (r ≈ +0.20) and Lequesne index (r ≈ +0.34), indicating that greater pain and disability relate to slower TUG performance.
- CADENCE was inversely associated with TUG (higher cadence corresponding to faster TUG performance), with a small-to-moderate effect size of approximately r ≈ −0.25.
- WOMAC showed a small positive association with VAS (r ≈ +0.19) and a moderate positive association with Lequesne index (r ≈ +0.30 to +0.50), reflecting overlapping constructs of pain and disability.
- Lequesne Index demonstrated a small-to-moderate positive association with VAS (r ≈ +0.31 to +0.35), consistent with their shared linkage to symptom severity and age.

Age and functional severity (pain and disability) show the clearest relationships with physical performance tests: higher pain/disability relates to shorter 6MWD and slower TUG, generally with small-to-moderate r values. Anthropometry has a modest influence.

QMI showed a small positive association with BMI (r ≈ +0.28) and with disability/pain scales (r ≈ +0.16 for VAS, r ≈ +0.18 for WOMAC, r ≈ +0.20 for Lequesne index) and small negative associations with ambulatory performance metrics: 6MWD (r ≈ −0.10) and CADENCE (r ≈ −0.14).

We conducted correlations only at baseline because they provide a clean snapshot of the natural associations among parameters, which is useful for understanding the mechanisms that condition gait patterns.

Post-intervention correlations may reflect effects of the rehabilitation program rather than intrinsic relationships between variables. Therefore, we limited correlations to the baseline assessment and reserved the final assessment for analyses on changes.

## 4. Discussion

### 4.1. Anthropometric Data

In our study, we monitored the effectiveness of gait retraining in KOA older adults, after the rehabilitation program, as well as the correlations between the quadriceps muscle dimension corrected for patient height and the other functional and physical performance parameters.

The average age for the two groups was approximately similar, 70 years (69.69 years in SG, 70.58 years in CG)—classifying the patients as young–old (between 65 and 74 years). This result is similar to the one specified in the literature that more than 50% of persons aged > 65 years suffer from some forms of arthritis [36], and KOA is one of the main causes of disability and affects the quality of life and economic status of patients [37].

In both patient groups, women were dominant. Worldwide, sex differences exist in the incidence rates of KOA [1]. The causes of this difference in KOA incidence remain unsolved and may be associated with estrogen levels.

We focused on the physical dimensions, both rehabilitation programs (physical activity/kinetic program), and the performances (physical function/physical performance-based measures) of our patients to define the real aspect of functioning and gait in KOA patients.

Complementary to mortality and morbidity, functioning is the third health indicator, and walking is an essential aspect of everyday life. The knee joint is the most complex joint of the human body and bears the greatest load among all joints. In 2020, Morais et al. mentioned that appropriate mechanical loading of joints is an important factor for joint health in general and knee joint health in particular. Similarly, we referred to KOA from the point of view of mechanical joint abnormalities. Long-term unloading of joints [38] has been shown to have negative effects on many tissues comprising the joints and represents a distinct risk for the development of KOA.

We established BMI and took into consideration its significant mechanical impact on lower-limb joints. BMI and significant knee pain can both contribute to poorer compliance with the kinetic program [39]. The patients did not have excessive BMI values, with a mean BMI of 26.26 for SG and 26.62 for CG (overweight). This supported the optimal delivery and adherence to the kinetic program for all participants.

### 4.2. Ultrasound Exam

The quadriceps femoris is the principal extensor of the knee and also contributes to hip flexion. By generating knee extension torque and stabilizing the patellofemoral and tibiofemoral joints, it plays a central role in knee (genu) mechanics, dynamic stability, and upright posture during gait and functional tasks.

Strengthening and improving quadriceps function lowers the risk of knee osteoarthritis (KOA), helps relieve pain in individuals with KOA, enhances mobility and task performance (e.g., sit-to-stand, stair climbing), and ultimately improves quality of life [14].

In KOA patients, ultrasound imaging of muscle provides clear visualization of key architectural features—such as cross-sectional area, thickness, fascicle length, and pennation angle [40]. By measuring echo intensity in KOA using ImageJ (Windows 11)—expressed as the mean pixel intensity within a defined region of interest—muscle changes can be monitored [41]. It can also quantify proportional changes in muscle composition and structure indirectly.

In 2017, Nunez and colleagues mentioned that a higher percentage of quadriceps muscle and better muscle quality (lower EI) was associated with better function in KOA patients [42].

We considered only muscle thickness, which was the parameter feasible to assess with our available ultrasound equipment. Muscle thickness evaluation is conducted with greater accuracy for the rectus femoris and vastus medialis, following established examination protocols. The argument for the significance of the vastus medialis is provided by Hoki and colab. The vastus medialis shows knee-structure-specific associations likely because of its distinct biomechanical role in stabilizing the joint, particularly by helping maintain medial patellar tracking under load. Its anatomical proximity to the medial tibiofemoral compartment also raises the possibility of local paracrine interactions or molecular crosstalk between intramuscular adipose tissue and adjacent cartilage [43].

The mean quadriceps muscle thickness in the studied patients (29.25 mm) is somewhat lower than the values reported in the literature (34.7 ± 9.5 mm, Núñez, M [42]).

Our findings are in line with values observed in cohorts of patients with KOA and concomitant sarcopenia (29.1 ± 5 mm in men, 25.3 ± 4.4 mm in women [44], 29.7 ± 7 mm in men, and 26.2 ± 7.1 mm in women [45]. However, we cannot definitively classify our patients as sarcopenic, since no formal sarcopenia assessment was conducted, despite their advanced age.

Ultrasound assessment was omitted at T2 as a minimum of 6 weeks of targeted exercise training is required to observe meaningful changes in muscle morphology and recovery of muscle parameters. The absence or weakness of correlations between quadriceps thickness—specifically between the chosen index and functional or performance parameters—does not invalidate the utility of ultrasound. Thickness is a simple morphologic indicator and is insufficient to capture the major determinants of performance in knee osteoarthritis (KOA), such as tissue quality (echogenicity), architecture (pennation, fascicle length), neuromuscular activation, pain, joint biomechanics (alignment, effusion, osteophytes), and comorbidities (systemic sarcopenia, neuropathy, obesity) [46]. Thickness alone misses key aspects of muscle quality, architecture, activation, and context. A more comprehensive assessment is achieved by combining imaging, strength, neuromuscular, and clinical measures, as we did in our study.

More advanced ultrasound measures (normalized echogenicity, elastography, cross-sectional area, vastus medialis/lateralis ratio, patellar tendon thickness) and functional assessments (e.g., isometric strength, TUG, 5xSTS, gait speed) should be interpreted in a complementary manner. Quadriceps ultrasound thus becomes a key link for: early detection of muscle degeneration, monitoring treatment response (exercise, weight loss, injections), tailoring rehabilitation programs (targeting vastus medialis, patellofemoral control), and explaining performance heterogeneity among patients with the same radiographic grade [47].

### 4.3. Rehabilitation Program

All patients performed a rehabilitation program adapted to individual functional status. We considered that the physical measures and kinetic program represent the optimal choice for KOA patients; moreover, the studied patients had comorbidities.

Our results reflect the data found in the medical literature, according to which, when applying an individualized kinetic program based on the severity of the disease, age, gender, and the individual’s functional status, pain relief and functional improvement are achieved in patients with KOA [48,49]. There is high-quality evidence demonstrating the effectiveness and the clinical benefits of therapeutic exercise regimens to improve pain, physical function, and quality of life in individuals with knee OA [49,50]. The evaluation and correction of gait in older adults with KOA are essential because they provide integrated information on pain, function, risk, and treatment response. Functionally, gait reflects the real capacity to carry out daily activities (independence, speed, endurance).

Classical exercise training (strength, flexibility, and endurance exercises) in KOA patients has only a modest impact on the motor control of walking. So, we included in our physical program special exercises for balance and gait retraining in SG patients. Balance exercises are important in KOA because this type of kinetic program improves the ability to control and stabilize body position and reduces the risk of falls in patients with KOA. Although the low quality of evidence addressing the use of balance exercises necessitates only a conditional recommendation for balance exercises [51], we consider that these exercises have a real benefit in gait retraining in our SG patients.

We performed visual biofeedback (mirror-guided biofeedback) and toe-out gait modification (to correct the altered foot progression angle), emphasizing equal cadence. These gait-retraining strategies appear to have strong evidence for effectively modifying walking biomechanics, as Rynne et al. mentioned in their review [51]. In 2020, Wang S et al. concluded in their systematic review that toe-out gait reduced mechanical knee loading in patients with mild-severe KOA [52,53]. In these two meta-analyses, it was concluded that the two gait-retraining strategies (real-time biofeedback and toe-out gait) demonstrated significant reductions in the knee adduction moment, with effect sizes of SMD −1.10 (95% CI −1.85 to −0.35) [51] and SMD −0.53 (95% CI −0.75 to −0.31) [52,53], respectively.

There is no consensus for clinicians as to which gait retraining strategies and tools are most effective for improving gait biomechanics and symptoms [54]. Regardless of the type of recommendations for gait retraining, our results confirmed the real benefit of the complete rehabilitation program in KOA.

Reduced walking speed and increased time for turning/initiation are associated with functional decline, hospitalizations, and increased mortality in older adults. So, KOA patients are encouraged to maintain a normal gait cadence. Increasing cadence, rather than stride length, would likely impose fewer alterations on lower limb biomechanics, particularly on the frontal plane knee moment, which is important in the progression of medial KOA [55,56]. After the rehabilitation program, our SG patients had improved their cadence by 9%. In the T2 moment, the mean value was 101.69 steps/minute. This result is in accordance with literature data [57].

The complete management of the KOA patient includes both kinetic and physiotherapy measures. Clinical application of physiotherapy improves blood circulation, provides anti-inflammatory and analgesic effects, and assists in the alleviation of symptoms [58,59]. For our research, we used electrical stimulation, electromagnetic therapy, laser therapy, and therapeutic ultrasound. These physiotherapy measures are the most commonly used by physical therapists in the rehabilitation of KOA patients. TENS was shown to improve pain and physical function in KOA patients in a 2009 study by Rutjes et al. [60]. In our study, TENS was applied not alone, so we could not consider that the effects were not produced only by the application of this physical procedure. We associated low-level laser therapy and ultrasound. As noted in other studies, the combination serves to limit pain and disability in patients with knee OA when compared to placebo [61,62].

### 4.4. Physical Performance and Gait Analysis

We applied both objective performance-based measures (6MWT, TUG) and patient-reported or self-reported measures (VAS, WOMAC, and Lequesne Index). It is important to assess what patients can do and what they perceive they can do. These two types of measures cannot be substituted for each other.

We have chosen only two performance-based physical tests (6MWT and TUG) from the core set of tests recommended by Osteoarthritis Research Society International (OARSI) [28].

We considered that gait, balance, and functional abilities that would be required for basic activities of daily living in our KOA patients should be optimally assessed with the 6MWT and TUG tests.

In our research, patients were tested using G-WALK. It consists of the inertial sensor G-SENSOR, the G-Studio software 2.8.16.0, and a set of protocols for the analysis of specific movements and for the gait spatio-temporal parameters [63]. The benefits of this sensor-based motion analysis are recognized in research on gait analysis [64].

In particular, we studied one global gait parameter—cadence (steps/min, number of half-steps in one minute) and SI (Symmetry Index). This index represents the subject’s ability to accelerate the center of mass in a similar way during the cycle of the right and left steps. The more the index approaches the value 100, the more symmetry there is during the path. Generally, non-pathological subjects show an index greater than 90. Before the rehabilitation program, the Symmetry Index values were close to 90: the mean was 88.53 for the study group (SG) and 92.30 for the control group (CG). After the intervention (T2), the mean increased to 92.95 in SG (+4.99%) and decreased to 90.64 in CG (−1.80%). Notably, the favorable progression was significant in the SG patients, who also underwent gait training, supporting its beneficial effect on gait coordination

The 6MWD assesses endurance and dynamic balance when changing directions during the walking activity, as well as the aerobic capacity and long-distance walking activity. In our study, the difference between T2 and T1 values was 59 m for SG and 13 m for CG. The SG patients were higher than a substantial MCID of 50 m, as has been estimated for the test in a sample of community-dwelling older adults with mobility dysfunction [65].

The TUG is considered a sensible test with an adequate minimal detectable change for clinical use only in KOA patients with I to III KL scoring, as were our patients. There is a need to test the validity of the OARSI-recommended physical tests in mild KOA patients [66]. We considered that TUG provides correct information for stability in walking, balance, moving from sitting to standing, and gait course changes. For the MCID, our results were 2.73 for SG patients and 0.84 for CG patients. The value for gait-retraining patients respects a recommended reduction of 0.8–1.4 s in OA research [26].

The patient-reported measures in this study were three of the most used scales in rehabilitation research—VAS, WOMAC, and Lequesne Index. These scales measure their psychometric properties for KOA.

The purpose of VAS is to measure pain. A pain reduction of 1.75 cm on the scale is the recommended MCID minimal clinically important difference in OA research [67]. We obtained 1.74 in SG patients.

WOMAC is a self-report questionnaire designed to assess the problems experienced by individuals with lower-limb osteoarthritis in the past 72 h. An improvement greater than or equal to 12% from baseline is the recommended MCID in OA research [66]. The MCID for the Lequesne Index is still not established in knee OA research [66]. Our SG patients showed improved functional status on the WOMAC scale, achieving an MCID of 12.98%, compared with only a 5% improvement in the CG.

Limitations. Our interpretation is constrained by the short follow-up period and the absence of longitudinal ultrasound assessments, which limits inferences about durability and mechanistic change over time. Six limitations should be addressed.

First, the assessments were conducted without blinding, which may introduce performance and detection bias. We implemented mitigation measures, including standardized procedures, pre-specified protocols/analyses, and evaluator training. We identify blinding as a priority for future studies.

Secondly, in our study, we assessed the functioning of KOA patients only with objective performance-based measures (6MWT, TUG) and patient-reported or self-reported measures (VAS, WOMAC, and Lequesne Index), but not according to the ICF model. This model is the result of an interaction among a person’s health condition, environmental factors, and personal factors [66], which were not measured in detail in the current study.

Third, we did not capture the global impact (pain, energy, and cognition), as patients were not evaluated using appropriate scales. It is well known that painful gait increases energy expenditure and fatigue, reduces physical activity, and promotes depression and cognitive decline. Nevertheless, the results obtained, showing improved gait in the study group, support an improvement in quality of life, possibly through reduced energy expenditure and a favorable effect on cognitive status.

Fourth, the 4-week post-intervention assessment (T2) was scheduled for logistical reasons, and adherence during this interval was not monitored, which limits inferences about the durability of effects; we acknowledge this as a limitation and include a sensitivity analysis to assess potential impact.

Fifth, no KOA patient has mild disease. In the future, we may extend the research to all stages of KOA.

Finally, we did not perform an ultrasound evaluation of the patients at the T2 time point. Participants received 2 weeks of supervised kinetic training, with instructions to continue at home thereafter; during this period, strict monitoring was not maintained. We therefore consider that the insufficient time interval and lack of adequate monitoring over the 4-week period justify our approach. In KOA, functional performance emerges from muscle quality, neuromuscular activation, pain, joint biomechanics, and comorbidities. Quadriceps thickness is a simple morphological metric that fails to reflect these dominant determinants, so poor or absent correlations with function and performance are not surprising. Future studies will include dynamic, longitudinal assessment of quadriceps morphology in this patient cohort. We selected muscle thickness for feasibility and reproducibility in our setting; however, the absence of echogenicity, pennation angle, and cross-sectional area measurements limits our assessment of muscle quality, a limitation now stated explicitly in the manuscript, and we propose including these measures in future studies. In this study, we derived a height-normalized quadriceps thickness index to minimize body-size influence and enable fair comparisons across subjects. The index is simple to implement, reproducible with minimal equipment (ultrasound and stadiometer) and shows low variability when using consistent anatomical landmarks and a standardized protocol. QHNI serves as a practical biomarker of quadriceps morphology in elderly patients with bilateral knee osteoarthritis. Because it is a structural proxy rather than a direct measure of muscle function, we limited our work to baseline ultrasound assessment. Longitudinal interpretation should be complemented by objective strength and performance testing to capture true functional change.

## 5. Conclusions

In older adults with knee osteoarthritis, the rehabilitation program should be preceded by a comprehensive baseline assessment that includes physical status, gait, and motivation, complemented by performance-based tests.

Our results support the utility of multimodal interventions (physical therapy, therapeutic exercise, gait training, pharmacotherapy, and health education); however, additional evidence and systematic syntheses are needed to establish their status as standards of care.

Gait training can reduce disability in KOA by improving balance control and gait pattern; clear walking recommendations are essential to optimize biomechanics and symptoms.

In the context of this study and the parameters used (muscle thickness), ultrasound measurement did not show a cross-sectional association with function. The absence of simple thickness–function correlations reflects KOA’s multifactorial limitations and supports a qualitative, integrated ultrasound profile rather than abandoning muscle imaging. The morphological index is a scalable, low-cost proxy for tracking atrophy/hypertrophy. While structural (not functional), it is useful for risk stratification when paired with gait metrics and functional tests. We underscore the practicality/feasibility of ultrasound for monitoring, but acknowledge that its utility as a functional marker requires additional muscle quality measures (echogenicity, pennation angle, cross-sectional area) and convergent validation in future studies.

## Figures and Tables

**Table 1:** Physical therapy applied for all patients.

Description	
Magnetic therapy BTL-5920 Czech Republic BTL Industries (Prague, Czech Republic) 10 sessions	Application with two coils (cervical/lumbar) and two cuboids at the knee level, latero-lateral. Series of rectangular magnetic pulses: pulse duration 20–300 ms, intensity 100 mT–28 mT/40 mT, 30 min per session per day.
TENS Endomed 482 device series 42.400, Enraf-Nonius, (The Netherlands) 10 sessions	Symmetric/asymmetric waveform, lateral knee region. Duration: 10–400 µs, in 5 µs increments; frequency: 1–200 Hz, in 1 Hz increments; modulation (spectrum): 0–180 Hz, in 1 Hz increments; modulation program: 1/1, 6/6, 12/12, 1/30/1/30 s; amplitude: 0–140 mA 20 min per session per day.
Ultrasound Chinesport Komby EL12059 10 sessions	EL0019 applicator, 5 cm^2^, peripatellar. Frequency: 1 MHz and 3 MHz ±15%. Adjustable duty cycle: 10–100%. Duty cycle frequency: 10–100 Hz. Maximum continuous/pulsed power: 0.6–1 W/cm^2^ ± 20%. Duration: 8 min per session per day
Low-level laser Astar PhysioGo 500I/501I, Poland PhysioGo series 10 sessions	400IRV3 applicator, 6 knee points (5 Joules per point) Laser radiation in continuous and pulsed modes, in visible and invisible ranges Low-level laser: 450 mW/808 nm 12 min per session per day

**Table 2:** Kinetic program.

**Kinetic Objectives**	**Description**
***5 min warm-up***	Free walking with arm swing.
**Conventional kinetic measures**	
Increase active knee flexion/extension	Passive movement of the lower limbs. Daily, 5 sets for each lower limb joint, distal to proximal, 10 min.
	Stretching calf, hamstring, and quadriceps muscles. Daily, 5 sets of 6–8 s for each muscle group.
Improving muscle strength and reducing the load on the symptomatic joint compartment	Calf muscles (leg flexors/extensors). Isotonic contraction, from orthostatism. Quadriceps muscles (vastus medialis). Hamstring muscles. Isotonic contraction, from shortened sitting. Gluteus medius muscle. Isotonic contraction, supine and antigravity. Daily, in an anti-gravity position for each muscle, 2 sets, 10 repetitions/set, with rest time corresponding to the duration of one set. Intensity equal to maximum voluntary contraction.
**Gait training**	
Postural control	Frenkel-type exercises, with voluntary movements performed without changing posture, 2 days a week, 30 min/session. Balance platform exercises. Proprioception exercises (Kabat diagonals, contraction/relaxation, agonist inhibition), 2 days a week, 30 min/session.
Balance exercises	
Gait coordination (maintaining alignment)	Front step and back step, over. Tandem walking. Modifying the toe-out gait. Mirror biofeedback. 3 days a week, alternating with previous sessions. 30 min/session
***10 min cool-down***	Active stretching for the hamstrings, thigh adductors, and triceps surae muscles
For relative rest, the patient was asked to maintain correct posture, alternating the position (with knees slightly flexed) with the functional position (with knees extended). Learning and observing orthopedic knee hygiene completed the program.	

**Table 3:** Study group. Demographic data and studied parameters (physical performance and functional status).

SG (Study Group)	Total	Women	Men	Urban	Rural
*n*	58	39 (67%)	19 (33%)	34 (58%)	24 (42%)
Age (years)	69.69 ± 4.52	69.72 ± 4.3	69.63 ± 5.08	70.21 ± 4.39	68.96 ± 4.7
BMI (kg/m^2^)	26.26 ± 2.66	27.47 ± 2.23	23.78 ± 1.49	26.72 ± 2.61	25.61 ± 2.65
QMI	21.42 ± 9.72	20.93 ± 11.77	22.43 ± 2.23	22.15 ± 12.55	20.39 ± 2.49
6MWD1 (m)	334.88 ± 59.19	337.56 ± 64.51	329.37 ± 47.58	335.15 ± 63.75	334.5 ± 53.41
6MWD2 (m)	393.62 ± 61.65	399.1 ± 65.11	382.37 ± 53.73	396.03 ± 64.28	390.21 ± 58.9
Cadence1 (steps/min)	92.57 ± 12.8	92.26 ± 11.98	93.21 ± 14.67	93.62 ± 12.99	91.08 ± 12.65
Cadence2 (steps/min)	101.69 ± 12.22	101.59 ± 11.76	101.89 ± 13.44	103.0 ± 12.71	99.83 ± 11.5
TUG1 (s)	20.37 ± 5.03	20.39 ± 5.17	20.34 ± 4.85	20.67 ± 5.41	19.96 ± 4.52
TUG2 (s)	17.64 ± 4.19	17.69 ± 4.17	17.54 ± 4.34	17.71 ± 4.44	17.54 ± 3.9
Symmetry Index1	88.53 ± 8.63	88.81 ± 6.61	87.96 ± 11.97	89.79 ± 7.24	86.75 ± 10.19
Symmetry Index2	92.95 ± 6.87	93.75 ± 3.63	91.32 ± 10.84	93.65 ± 4.29	91.96 ± 9.42
VAS1	6.83 ± 0.96	6.69 ± 0.98	7.11 ± 0.88	6.65 ± 0.88	7.08 ± 1.02
VAS2	5.09 ± 0.76	5.03 ± 0.78	5.21 ± 0.71	4.97 ± 0.72	5.25 ± 0.79
Lequesne Index1	9.59 ± 1.8	9.41 ± 1.82	9.95 ± 1.76	9.71 ± 1.82	9.42 ± 1.8
Lequesne Index2	7.6 ± 1.31	7.54 ± 1.41	7.74 ± 1.1	7.74 ± 1.39	7.42 ± 1.18
WOMAC1	65.59 ± 8.04	64.64 ± 8.42	67.53 ± 7.03	65.03 ± 8.37	66.38 ± 7.67
WOMAC2	57.07 ± 5.96	56.49 ± 6.32	58.26 ± 5.09	56.44 ± 5.25	57.96 ± 6.87

Data are presented as mean ± SD for continuous variables and *n* (%) for categorical variables.

**Table 4:** Control group. Demographic data and studied parameters (physical performance and functional status).

CG (Control Group)	Total	Women	Men	Urban	Rural
*n*	62	38 (61%)	24 (39%)	30 (48%)	32 (52%)
Age (years)	70.58 ± 4.62	70.08 ± 4.8	71.38 ± 4.3	70.07 ± 4.69	71.06 ± 4.58
BMI (kg/m^2^)	26.62 ± 2.92	28.07 ± 2.37	24.33 ± 2.14	27.37 ± 2.78	25.92 ± 2.91
QMI	21.06 ± 2.75	19.92 ± 1.82	22.87 ± 3.01	20.31 ± 2.76	21.77 ± 2.58
6MWD1 (m)	316.31 ± 43.9	322.29 ± 47.33	306.83 ± 36.82	322.13 ± 44.57	310.84 ± 43.25
6MWD2 (m)	329.48 ± 37.96	332.74 ± 39.33	324.33 ± 35.9	329.8 ± 38.74	329.19 ± 37.83
Cadence1 (steps/min)	91.08 ± 13.62	90.63 ± 13.55	91.79 ± 13.99	89.3 ± 13.42	92.75 ± 13.8
Cadence2 (steps/min)	89.71 ± 13.07	89.55 ± 12.42	89.96 ± 14.31	88.47 ± 13.37	90.88 ± 12.88
TUG1 (s)	24.45 ± 4.56	24.3 ± 4.9	24.69 ± 4.05	24.03 ± 4.9	24.85 ± 4.25
TUG2 (s)	23.61 ± 4.99	23.12 ± 5.18	24.38 ± 4.67	22.97 ± 5.47	24.21 ± 4.5
Symmetry Index1	92.3 ± 5.67	91.83 ± 6.23	93.04 ± 4.69	92.06 ± 6.23	92.52 ± 5.18
Symmetry Index2	90.34 ± 5.98	90.43 ± 6.27	90.19 ± 5.62	90.24 ± 5.5	90.42 ± 6.48
VAS1	6.56 ± 0.84	6.55 ± 0.89	6.58 ± 0.78	6.53 ± 0.82	6.59 ± 0.87
VAS2	6.63 ± 0.63	6.66 ± 0.63	6.58 ± 0.65	6.67 ± 0.66	6.59 ± 0.61
Lequesne Index1	11.31 ± 1.77	10.88 ± 1.83	11.98 ± 1.48	11.12 ± 1.97	11.48 ± 1.58
Lequesne Index2	10.82 ± 1.34	10.62 ± 1.37	11.15 ± 1.25	10.6 ± 1.5	11.03 ± 1.15
WOMAC1	58.61 ± 5.37	57.89 ± 5.29	59.75 ± 5.41	57.87 ± 5.25	59.31 ± 5.47
WOMAC2	55.65 ± 4.57	55.34 ± 4.7	56.12 ± 4.4	55.1 ± 4.25	56.16 ± 4.86

Data are presented as mean ± SD for continuous variables and *n* (%) for categorical variables.

**Table 5:** The studied parameters and statistical analysis (between-group comparisons).

Group	Measure	*n*	Baseline (Mean ± SD)	Post (Mean ± SD)	Delta (95% CI)	*p*-Value	Effect Type	Effect Size
Study	6MWD	58	334.88 ± 59.19	393.62 ± 61.65	58.74 (49.246 to 68.236)	<0.0001 (4.89 × 10^−10^)	r	0.817
Study	TUG	58	20.13 ± 4.91	17.52 ± 3.97	−2.61 (−3.113 to −2.108)	<0.0001 (5.31 × 10^−11^)	r	0.862
Study	CADENCE	58	92.57 ± 12.8	101.69 ± 12.22	9.12 (7.587 to 10.654)	<0.0001 (6.88 × 10^−11^)	r	0.857
Study	SI	58	88.53 ± 8.63	92.95 ± 6.87	4.42 (3.116 to 5.722)	<0.0001 (3.93 × 10^−8^)	r	0.721
Study	VAS	58	6.83 ± 0.96	5.09 ± 0.76	−1.74 (−1.936 to −1.547)	<0.0001 (3.26 × 10^−11^)	r	0.871
Study	WOMAC	58	65.59 ± 8.04	57.07 ± 5.96	−8.52 (−9.738 to −7.297)	< 0.0001 (4.65 × 10^−20^)	r	−0.183
Study	Lequesne Index	58	9.59 ± 1.8	7.6 ± 1.31	−1.98 (−2.237 to −1.728)	<0.0001 (3.88 × 10^−11^)	r	0.868
Control	6MWD	62	316.31 ± 43.9	329.48 ± 37.96	13.18 (6.029 to 20.326)	0.000486	r	0.468
Control	TUG	62	24.45 ± 4.56	23.61 ± 4.99	−0.84 (−1.263 to −0.419)	0.000194	r	0.473
Control	CADENCE	62	91.08 ± 13.62	89.71 ± 13.07	−1.37 (−3.274 to 0.532)	0.000573	r	0.437
Control	SI	62	92.3 ± 5.67	90.34 ± 5.98	−1.96 (−2.957 to −0.966)	<0.0001 (3.67 × 10^−6^)	r	0.588
Control	VAS	62	6.56 ± 0.84	6.63 ± 0.63	0.06 (−0.151 to 0.28)	0.543793	r	0.077
Control	WOMAC	62	58.61 ± 5.37	55.65 ± 4.57	−2.97 (−3.984 to −1.952)	<0.0001 (4.57 × 10^−7^)	r	0.641
Control	Lequesne Index	62	11.31 ± 1.77	10.82 ± 1.34	−0.48 (−0.749 to −0.219)	<0.0001 (5.71 × 10^−5^)	r	0.511

*p*-value established with the Mann–Whitney U test; higher r values indicate stronger effects.

## Data Availability

Data supporting reported results can be provided by the corresponding author on request.

## Abbreviations

The following abbreviations are used in this manuscript:

ACR	American College of Rheumatology criteria for KOA
BMI	body mass index
CG	control group
SG	study group
TUG	Timed Up-and-Go
SI	Symmetry Index
6 MWD	Six-Minute Walk Distance
6 MWT	Six-Minute Walk test
QHNI	height-normalized quadriceps thickness index
MRI	Magnetic Resonance Image
TENS	transcutaneous nerve stimulation
VAS	Visual Analog Scale
WOMAC	The Western Ontario and McMaster Universities Osteoarthritis Index
